# Perspective for elasticity of minerals in the Earth's top lower mantle

**DOI:** 10.1093/nsr/nwaa270

**Published:** 2020-10-29

**Authors:** Zhu Mao, Ningyu Sun, Wei Wei

**Affiliations:** Laboratory of Seismology and Physics of Earth's Interior, School of Earth and Space Sciences, University of Science and Technology of China, China; CAS Center for Excellence in Comparative Planetology, University of Science and Technology of China, China; Laboratory of Seismology and Physics of Earth's Interior, School of Earth and Space Sciences, University of Science and Technology of China, China; CAS Center for Excellence in Comparative Planetology, University of Science and Technology of China, China; Laboratory of Seismology and Physics of Earth's Interior, School of Earth and Space Sciences, University of Science and Technology of China, China

## Abstract

The elasticity of minerals under high pressure-temperature conditions is crucial for constraining mantle composition. This perspective highlights recent advances in experimental and theoretical work in determining the elasticity of minerals to resolve the origin of low-velocity layers and the seismic anisotropy in the top lower mantle

The lower mantle, ranging from 660 to 2891 km in depth, comprises the vast majority of Earth's volume. Although the Earth's lower mantle has been regarded as compositionally homogeneous in the first order and isotropic until 150–300 km above the core-mantle boundary, there is growing seismic evidence indicating a rather complex image of the top lower mantle at depths of 660–1200 km (Fig. 1) [1,2]. Beneath northeast Asia and North America, a 1.5–2.6% reduction in the shear-wave velocity was identified at depths of 660–800 km [1]. Near the major global subduction zones at depths of 660–1200 km, the SH wave (propagation direction parallel to the vibration direction) was observed as being up to 2% faster than the SV wave (propagation direction perpendicular to the vibration direction), showing the presence of seismic anisotropy in the top lower mantle [2]. Deciphering the formation mechanism of the low-velocity layer and the anisotropic structure requires detailed knowledge of the elasticity of minerals under relevant pressure-temperature (P-T) conditions of the lower mantle. However, it has been a long-standing challenge in both experimental and theoretical studies to determine the elasticity of lower-mantle minerals because of the extremely high P-T conditions of the region.

**Figure 1.**
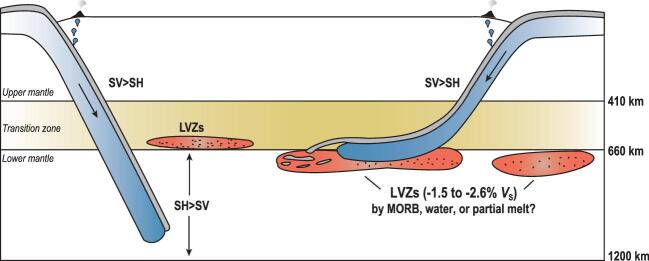
The schematic of the velocity anomalies in the top lower mantle. Low-velocity layers were observed beneath the stagnant slabs, and the location where no slab is present [1]. This can be explained as accumulation of MORB, water, or water-induced partial melt. The top lower mantle beneath the major subduction zone was identified to be anisotropic with a faster SH wave [2].

Recent progress in high-pressure techniques significantly advances our capability of constraining the elasticity of minerals under lower-mantle P-T conditions. Using a large-volume press, the elasticity of lower-mantle CaSiO_3_-perovskite was determined to be up to 23 GPa and 1700 K (∼630 km depth at 1700 K), showing a substantially low shear modulus of 126(1) GPa for CaSiO_3_-perovskite [3]. Although the volume percentage of CaSiO_3_-perovskite in the pyrolitic lower mantle is only 7–10%, it can be as high as 22–29% in the subducted mid-ocean ridge basalt (MORB). Accumulation of MORB in the top lower mantle with the presence of 23 vol.% low-velocity CaSiO_3_-perovskite will have a seismic signature consistent with the observed low-velocity layer in the top lower mantle [3]. However, a more complicated problem is that lower-mantle CaSiO_3_-perovskite could incorporate certain amounts of Al and Ti in the structure [4]. Although addition of Al and Ti has been noted to influence the phase transition between cubic and orthorhombic/tetragonal CaSiO_3_-perovskite [4], experimental and theoretical constraints on the effect of Al and Ti on the elasticity of CaSiO_3_-perovskite are lacking, which is important for tracking the existence of MORB and understanding the origin of the low velocity anomalies in the top lower mantle.

In addition, the presence of water or water-induced partial melting has also been invoked to explain the detected low-velocity layer in the top lower mantle beneath northeast Asia and North America [1]. Hydrous phases, such as superhydrous phase B, phase D and δ-AlOOH, could survive during slab sinking and be transported to the lower-mantle. Stishovite (SiO_2_), one of the dominant phases in the subducted MORB with a volume percentage of 20%, could also accommodate 3.2–10 wt.% water under lower mantle P-T conditions [5]. The accumulation of these hydrous phases has been speculated to lower the velocity of the top lower mantle substantially. However, sound velocities of these hydrous phases have been constrained only by elasticity data experimentally obtained at a high pressure of 300 K or under P-T conditions much lower than those in the top lower mantle. Theoretical predictions for the elasticity of these hydrous phases are limited, and direct measurements on the elasticity of hydrous stishovite are not available. In addition, the dehydration of hydrous minerals, such as hydrous ringwoodite under relevant P-T conditions in the top lower mantle, could induce partial melt because of a much lower water-storage capacity of lower mantle minerals [1]. The presence of partial melt can substantially lower the sound velocity [6]. However, experimental and theoretical studies on the sound velocity of water-induced partial melts are limited to upper mantle P-T conditions or simplified melt geometries [6]. Detailed knowledge of the elasticity of these hydrous phases and sound velocity of partial melts under the relevant P-T conditions of the top lower mantle could help to discriminate the water-rich regions from the remnants of the subducted oceanic crust.

Unlike the D^″^ layer (the bottom 100–300 km of the lowermost mantle) with strong anisotropy, the top lower mantle has long been considered to be radially isotropic in the shear wave. Until recently, a three-dimensional global seismic tomographic study observed the presence of anisotropy beneath subduction slabs in the top lower mantle with an SH wave 2% faster than the SV wave down to a depth of 1200 km [2]. In the mantle, seismic anisotropy is normally produced by the lattice preferred orientation (LPO) of anisotropic mantle minerals. Knowledge of the single-crystal elasticity of lower-mantle minerals together with the LPO determined by deformation experiments under high P-T conditions is key to deciphering the formation mechanism of the anisotropic structure and deformation history of the top lower mantle [7].

Bridgmanite [(Mg,Fe)(Al,Fe,Si)O_3_] is the primary constituent of the lower mantle, with a volume percentage of 75%. It is considerably difficult to determine the single-crystal elasticity of bridgmanite. It first requires synthesis of high-quality single crystals using a large-volume press at ∼24 GPa, which has become feasible in recent years. In addition, bridgmanite crystallizes in an orthorhombic structure with nine independent elastic constants and is the major host of Fe (Fe^2+^ and Fe^3+^) and Al. Its complex crystal structure and composition highlight the difficulty of both experimental measurements and theoretical calculations. To date, the single-crystal elasticity of bridgmanite under simultaneously high P-T conditions is only available for the MgSiO_3_ endmember from theoretical calculations [8]. None of the experiments were performed under simultaneous high P-T conditions. The combined effect of Fe and Al on the single-crystal elasticity of bridgmanite was studied only at high pressure and 300 K [9]. These elasticity data have not been applied in combination with deformation results to model the anisotropic structure of the top lower mantle.

Compared to bridgmanite, ferropericlase [(Mg,Fe)O] is the second most abundant mineral in the Earth's lower mantle. It crystallizes in the cubic structure with only three independent elastic constants but is highly anisotropic. Despite its simpler structure, the single-crystal elasticity of ferropericlase has been experimentally determined only up to 50 GPa at 900 K, which is at a much lower temperature condition than the Earth's mantle [10]. The current understanding of the anisotropic behavior of ferropericlase under relevant P-T conditions of the lower mantle relies heavily on theoretical calculations. Thus, there is an urgent need for systematic investigations of the single-crystal elasticity of bridgmanite and ferropericlase with varying compositions under simultaneous high P-T conditions.

In summary, the seismic structure of the Earth's top lower mantle is much more complex than previously thought. High-pressure studies on the elasticity of minerals under relevant P-T conditions of the top lower mantle are critical to resolving the observed obscure low-velocity layer and anisotropic structure in the region. Although significant progress has been achieved in experimental and theoretical investigations of the elasticity of minerals under high P-T conditions, future studies are expected to focus on the combined effect of pressure, temperature and composition variation on elasticity, particularly single-crystal elasticity.
